# Preoperative Prediction of Axillary Lymph Node Metastasis in Breast Cancer using Radiomics Features of DCE-MRI

**DOI:** 10.1038/s41598-019-38502-0

**Published:** 2019-02-19

**Authors:** Xiaoyu Cui, Nian Wang, Yue Zhao, Shuo Chen, Songbai Li, Mingjie Xu, Ruimei Chai

**Affiliations:** 1 Northeastern University, Sino-Dutch Biomedical and Information Engineering School, Wisdom Street, Shenyang, 110819 China; 2 Radiology Department, The first hospital of China Medical University, 155# North Nanjing street, Heping district, Shenyang, 110001 China

## Abstract

The accurate and noninvasive preoperative prediction of the state of the axillary lymph nodes is significant for breast cancer staging, therapy and the prognosis of patients. In this study, we analyzed the possibility of axillary lymph node metastasis directly based on Magnetic Resonance Imaging (MRI) of the breast in cancer patients. After mass segmentation and feature analysis, the SVM, KNN, and LDA three classifiers were used to distinguish the axillary lymph node state in 5-fold cross-validation. The results showed that the effect of the SVM classifier in predicting breast axillary lymph node metastasis was significantly higher than that of the KNN classifier and LDA classifier. The SVM classifier performed best, with the highest accuracy of 89.54%, and obtained an AUC of 0.8615 for identifying the lymph node status. Each feature was analyzed separately and the results showed that the effect of feature combination was obviously better than that of any individual feature on its own.

## Introduction

Breast cancer is the most common cancer diagnosed among US women (excluding skin cancers) and is the second leading cause of cancer death among women after lung cancer^1,2^. Breast cancer is often accompanied by axillary lymph node metastasis (ALNM). Whether ALNM is involved has great significance for breast cancer staging, therapy and prognosis of patients and is also one of the important reference indexes for postoperative radiotherapy and chemotherapy. To accurately understand the axillary lymph node (ALN) stage and to reduce the incidence of postoperative complications, it is very important to select a safe and effective treatment for the preoperative prediction and diagnosis of the ALN status^3^.

Clinically, the gold standard for assessing the metastasis of axillary lymph nodes in breast cancer usually includes axillary lymph node dissection (ALND) and sentinel lymph node biopsy (SLNB). ALND is a standard surgical approach for all patients in the 20th century to both assess ALN status and treat metastatic ALNs. It provides the most complete and accurate information; however, as many as approximately 70% of early breast cancer patients exhibit no ALN metastasis. In these cases, ALND can be deemed a significant overtreatment that is associated with significant trauma and many complications^4^. SLNB, an operation devised to reduce the need for ALND and a method of staging the axilla of patients with clinically negative axilla^5,6^, has been performed as a standard procedure in breast cancer surgery^7–9^. SLNB can be used to predict ALNM with high accuracy, and it precludes the removal of the ALNs and the subsequent complications associated with axillary clearance in node-negative breast cancer patients^7,10^.

However, SLNB is still an invasive procedure. Therefore, a series of predictive methods were suggested to screen patients with a low risk of ALNM and to avoid SLNB. Clinical examination, imaging examination, ultrasound-guided fine-needle aspiration and mathematical models are commonly used to predict ALNM status. Clinical examination is the most traditional and basic means of ALN examination; however, the doctor’s experience has a great impact on the results. Ultrasound, mammography, CT, MRI and other imaging examinations can improve the accuracy of prediction, but there are high false-negatives. Ultrasound-guided fine-needle aspiration of the ALNs can effectively improve the preoperative diagnosis rate. However, even if there is no evidence of cancer metastasis, further surgery is needed to determine the ALN staging. The mathematical model is a way to predict ALNM. Bevilacqua *et al*.^11^ performed a multifactor analysis on the clinicopathological factors of 3786 patients with SLNB. The results showed that the following 8 factors were significantly associated with ALNM: age, size of primary tumor, tumor type, vascular invasion, location, multifocal, ER and PR. The AUC value of 0.754 was validated in 1545 patients. The clinicopathological factors of 1395 patients were analyzed by Meretoja^12^ *et al*. The results showed that size, multifocal lesions, and vascular invasion were significantly related to ALNM, and the AUC was 0.750. Chen *et al*.^13^ and Qiu *et al*.^14^ used the MSKCC model to calculate the ALNM of 524 patients and 1227 patients to evaluate the clinical value of the model in early breast cancer in Chinese patients, and the AUC values were 0.757 and 0.730, respectively.

The above model of predicting ALNM is based on a retrospective analysis of the clinicopathological factors of breast cancer patients and the selection of statistically significant factors. The correlation between the tumor size, vascular invasion and the LNM is reflected in many models^11,12,15^. The AUC of most of the models was approximately 0.70, which was not associated with a very precise prediction result. In the study, radiomic signatures transform breast MRI into high-dimensional data by high-throughput quantitative feature extraction. In addition, then the SVM, KNN, and LDA three classifiers were used to achieve the purpose of preoperative prediction of ALNM and yielded the best accuracy of 89.54%.

## Methods

### Materials

This study was approved by the Ethics Committee of the First Hospital of China Medical University, without informed consent. A total of 115 breast MRI studies of 102 patients were obtained from the First Hospital of China Medical University. Of these imaging studies, 13 patients had two lesions; therefore, each lesion was treated as an individual patient. The ages of the patients ranged from 35 to 60 years, which means that the data set is a representative subset of all breast cancer patients. All patients were female who had been diagnosed with benign primary breast carcinoma. They underwent ALND or SLNB to obtain pathological results. According to the pathological results, the patients were divided into a metastasis group (n = 52) or a nonmetastasis group (n = 63).

All MR images were acquired by Siemens 3.0 T MRI scanning equipment and a dedicated breast phased-array coil. During the examination, the patient assumed the prone position with both hands flat on the sides of the body. The bilateral breasts were fully exposed and naturally fell into the breast phased-array coils. The acquisition parameters of the 3.0 T equipment were as follows: the pulse repetition time (TR) = 4.67 ms, the echo time (TE) = 1.66 ms, the layer scanning without interval, the layer thickness = 1.2 mm and the image matrix = 384 * 384. Enhanced contrast material, Gd-DTPA, was injected intravenously at a dose of 0.2 mmol/kg using a high pressure syringe at a rate of 2.0 ml/s, followed by the same rate of injection of 15 ml saline to wash the residual contrast agent in the tube. A total of 8 phases were scanned, and the first phase involved plain scanning. The injected contrast agent began to enhance the scan for a total of 7 phases. In this study, we used the 2nd post-contrast image sequences of enhanced scanning as the experimental data, because the image lesions in the second phase (60~120 s) had the largest amount of contrast with the background^16^.

### Data preprocessing

In this study, most of the image sizes are 384*384, but some of the image sizes are still 320*320 or 448*448. To facilitate the next processing, resampling is used to unify all image sizes to 384*384.

### Segmentation

The mass segmentation of the breast is the basic part of the method and is also the key step for the detection and diagnosis of the lesions. Current clinical physicians manually divide the lesion based on experience. However, due to the huge amount of data in the image sequence, there is a large amount of redundant information, which leads to the huge workload of manual segmentation and the existence of subjectivity. Therefore, in the study, to improve the diagnostic performance of the system and achieve the efficient segmentation function, we integrated the process of browsing the image into the segmentation process, providing an interactive and semiautomatic segmentation method for the lesions in the breast sequence.

Regional growth is one of the image segmentation techniques. The basic idea of regional growth is to assemble pixels with similarity to form a region. First, each region is divided to find a seed pixel as the starting point of growth, and then the pixels with the same or similar properties (similarity criteria according to predetermined growth) are combined to form the region. The region then merges with the neighborhood of the seed pixel. The new pixels continue to grow similarly to the seed pixels in the area until the pixels that do not meet the conditions can be included, and a region grows. The original images input in the experiment are 16-bit data, so we use “im2double” function to normalize the image to [0,1] before the regional growth algorithm is used to segment the mass.

Therefore, in the study, the segmentation process is mainly divided into the following four steps:

1. The doctor browses the 2nd post-contrast image sequences of enhanced scanning, selects one tomographic image containing the markedly enhanced lesion, and marks the lesion area on the image.
2. According to the general outline of the lesion marked by the doctor, a rectangular ROI containing the lesion is drawn to reduce the computational complexity. The surrounding tissues may be selected when the threshold of the regional growth is larger. The rectangular ROI is automatically constructed for other images according to the drawn image.
3. Within the selected ROI, a seed point is manually selected. The volume data is segmented according to the regional growth segmentation algorithm.
4. To objectively reflect the segmentation effect, the Dice coefficient and ICC of the feature were used to quantitatively analyze the segmentation results.

### Feature Extraction and Selection

Feature extraction is an important basis for quantifying and expressing image information. Correct extraction and selection of effective radiomic signatures is an important part of determining axillary lymph node status and is also an important prerequisite to ensure classification accuracy and diagnostic effect.

In this study, we extract 2 types of features, namely, morphological features and texture features. Features are extracted based on all images of segmented masses. The feature set describing each mass included 58 features as follows: 16 morphological features, 8 features extracted by the Gray Level Co-occurrence Matrix (GLCM, also known as Haralick features)^17^, 5 features extracted from the Gray Level Run Length Matrix (GLRLM), 15 features extracted from the Gray Level-Gradient Co-occurrence Matrix (GLGCM), 5 features extracted from the Neighboring Gray-Level Dependence Matrix (NGLDM), 3 features extracted by Tamura, and 6 grayscale histogram features. Among them, GLCM, GLRLM, GLGCM, NGLDM, grayscale histogram features, and Tamura features are all texture features. These features are presented in detail in Appendix 1. In all feature extraction except for GLCM, the number of gray levels is 256. In GLCM feature extraction, the number of gray levels is 16.

Feature selection plays an important role in training classifiers, reducing the computational complexity and improving the classification accuracy. The least absolute shrinkage and selection operator (LASSO) method, which is suitable for the regression of high-dimensional data^17,18^, is used to select the most useful predictive features from the primary data set. The basic idea of LASSO is to minimize the residual square sum under the constraint that the sum of the absolute values of the regression coefficients is smaller than a constant, so that some regression coefficients that are strictly equal to zero can be generated.

### Classification

In the prediction system of ALNM based on breast MRI, the characteristic parameters obtained after lesion segmentation, feature extraction and selection must be input into the classifier to obtain the final lymph node metastasis prediction results, to achieve a complete lesion diagnosis.

This article discusses 3 kinds of typical classifiers: the SVM classifier, KNN classifier and LDA classifier, which currently are commonly used to predict ALNM of breast cancer. Part of our proposal is implemented and executed on MATLAB 2017a, and a nomogram was built using R software (version 3.3.4).

## Results

### Segmentation

**Figure 1:**
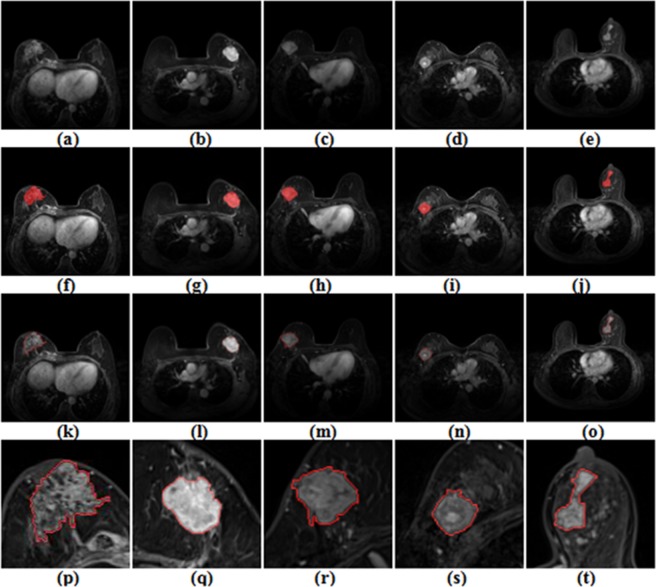
The segmentation results are shown. (**a**~**e**) is the original image containing the markedly enhanced lesion; (**f**~**j**) is the gold standard marked by the doctors; (**k**~**o**) is the approximate outline of the regional growth (red); and (**p**~**t**) is the enlarged view of the segmented lesion area (red).

The Dice coefficient of the gold standard and regional growth segmentation was 0.9011. This showed that the semiautomatic segmentation algorithm had a good effect and could be used for subsequent feature extraction. Based on the different independent segmentation groups, intraclass correlation coefficients (ICCs) were used to estimate each radiomic feature’s stability. The results showed that the ICCs of all features were greater than 0.8. Thus, they were used in the model construction. Segmentation results are shown in Fig. 1.

### Feature Extraction and Selection

**Figure 2:**
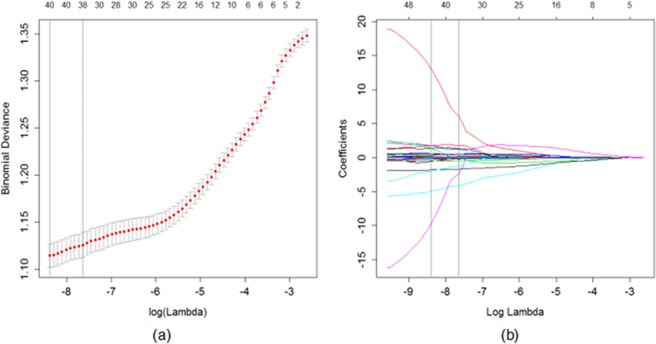
LASSO regression model for feature dimension reduction. (**a**) Selection of the parameter (λ) in the LASSO model by 10-fold cross-validation based on minimum criteria. The y-axis indicates binomial deviances. The lower x-axis indicates the log(λ). Red dots indicate the average deviance values for each model with a given λ, and vertical bars through the red dots show the upper and lower values of the deviances. The vertical black lines define the optimal values of λ, where the model provides the best fit to the data. A λ value of 0.00048, was chosen. (**b**) LASSO coefficient profiles of features. The dotted vertical line was plotted at the value selected using 10-fold cross-validation, where optimal λ resulted in 14 nonzero coefficients.

To reduce complexity, 58 features were reduced to 38 potential predictors on the basis of 115 patients in the LASSO logistic regression model. The 38 features include 14 morphological features, 1 NGLDM feature, 4 GLRLM features, 7 GLCM features, 10 GLGCM features, 1 Tamura feature, and 1 grayscale histogram feature. These features are presented in detail in Appendix 2. The feature selection process is shown in Fig. 2.

### Classification

Comparing original features with features selected by LASSO, the results of the three classifiers were shown in Table 1. Considering the results of Table 1 comprehensively, we could see that the accuracy and sensitivity of the SVM classifier in predicting breast ALNM were significantly higher than those of the KNN classifier and LDA classifier, and the overall effect of KNN classifier was better than that of the LDA classifier. After the feature dimension reduction, the accuracy of three classifiers were all slightly higher, from 88.24% to 89.54%, from 82.06% to 86.82% and from 70.99% to 74.27%, respectively. Therefore, in the experiment, 38 features extracted by LASSO could represent most of the information for classification. It also showed that the performance of the classification prediction model based on 38 features was better than that of the model based on all features.

**Table 1:** Predictive value of three classifiers based on all features and extracted features using the LASSO method.

Three Classifiers	38 features extracted by LASSO				58 features			
	Accuracy	Sensitivity	Specificity	AUC	Accuracy	Sensitivity	Specificity	AUC
SVM	89.54%	94.50%	80.06%	0.8615	88.24%	94.90%	77.96%	0.8658
KNN	86.82%	89.39%	87.18%	0.8436	82.06%	74.71%	73.36%	0.7041
LDA	74.27%	89.43%	50.67%	0.6367	70.99%	80.31%	67.78%	0.6837

**Figure 3:**
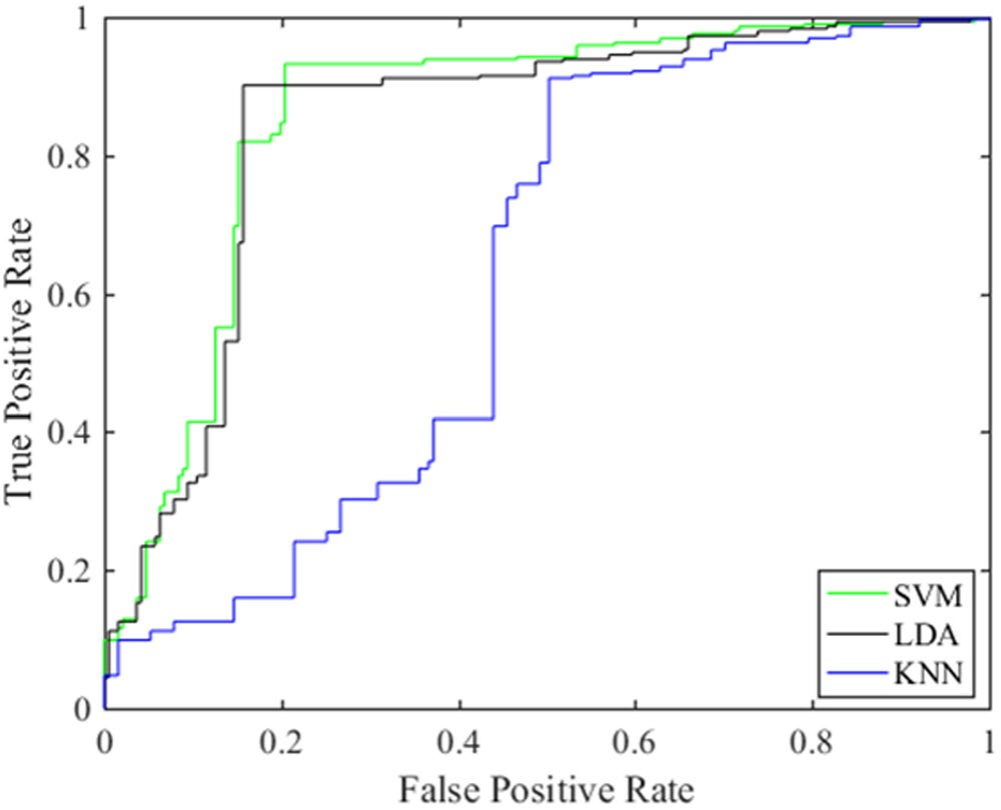
The AUC for the SVM, KNN and LDA classifiers.

The SVM classifier performed best with the highest accuracy of 89.54%. Whether the breast showed ALNM or not was also well recognized, with a sensitivity and a specificity of 94.50% and 80.06%, respectively. The AUC value of the SVM classifier was significantly higher than that of the KNN and LDA classifiers. The ROC curve of our proposal is shown in Fig. 3. In short, through the SVM classifier, MRI had a high predictive value for metastatic and nonmetastatic ALN. David. *et al*.^19^ obtained an AUC of 0.88 for the task of distinguishing between positive and negative nodes using the neural net classifier in the leave-one-case-out cross-validation based on MRI of the axillary lymph nodes.

**Figure 4:**
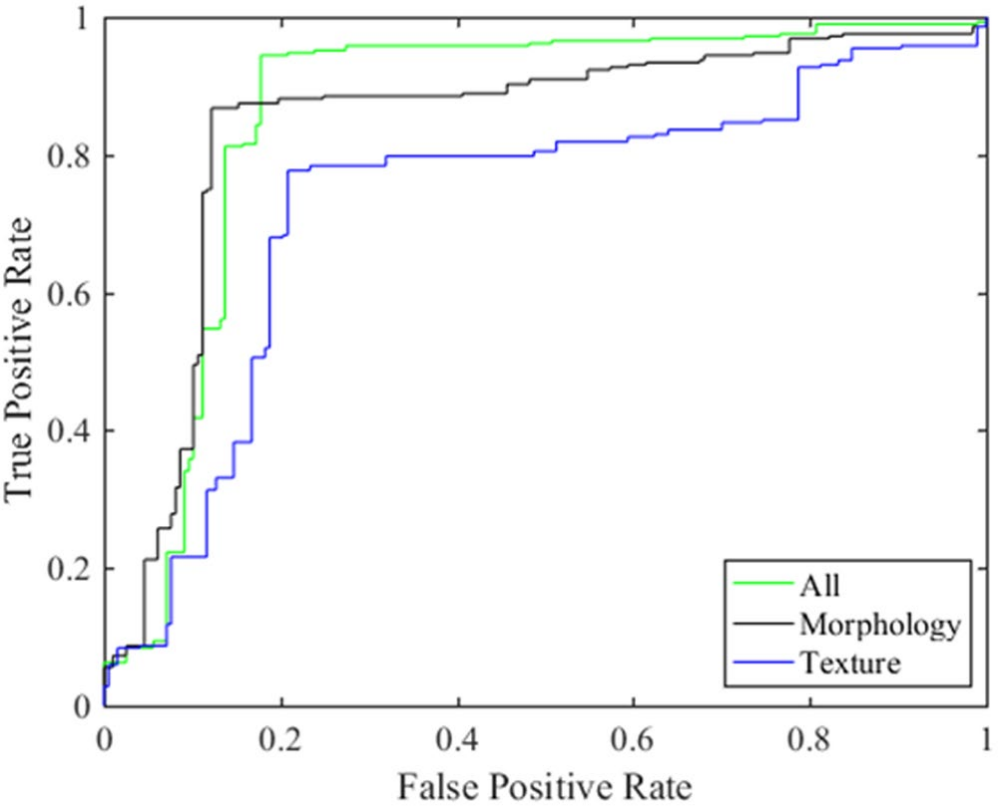
The AUC of the morphological features and texture features.

Based on the SVM classifier, 14 morphological features and 24 texture features selected by LASSO were used separately to build a classification prediction model. The analysis results were shown in Table 2 and Fig. 4. When using morphological features alone, the accuracy, sensitivity, specificity and AUC were 77.69%, 76.41%, 79.50% and 0.8421, respectively. When analyzing texture features separately, the accuracy, sensitivity, specificity and AUC were 85.78%, 90.37%, 76.00% and 0.7098, respectively. The results showed that whether morphological features or texture features were used alone, the effect was lower than that of the combination of morphological and texture features.

**Table 2:** Comparison of morphological features and texture features.

Features	Accuracy	Sensitivity	Specificity	AUC
38 feature	89.54%	94.50%	80.06%	0.8615
Morphology	77.69%	76.41%	79.50%	0.8421
Texture	85.78%	90.37%	76.00%	0.7098

**Figure 5:**
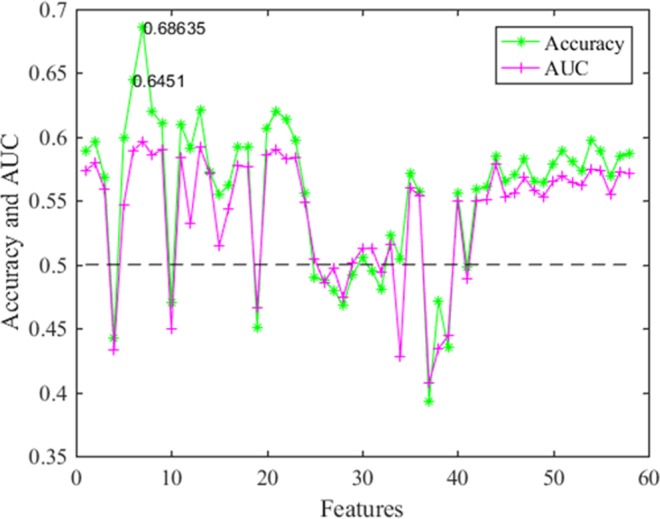
Results of the individual analysis of 58 features.

Single factor analysis was used to test the ability of 58 features to differentiate ALNM when used alone. The results were shown in Fig. 5. Among them, the serial numbers 1–16 represented the morphological features, and 17–58 were texture features. From the overall perspective of Fig. 5, the accuracies of the morphological features were superior to those of the texture features. The accuracies and AUCs of all morphological features were better than the baseline of random chance (=0.5) except for ratio of length to width and eccentricity of an ellipse with the same second-order moment as the mass area. However, the AUCs and accuracies of the serial numbers 25–40 were basically less than guessing. In all single feature analysis, the best accuracy was 68.72% and the second highest accuracy is 64.51%. They are abscissa and ordinate of centroid in morphological features. Results showed that the effect of feature combination was obviously better than the performance of any individual feature on its own.

**Figure 6:**
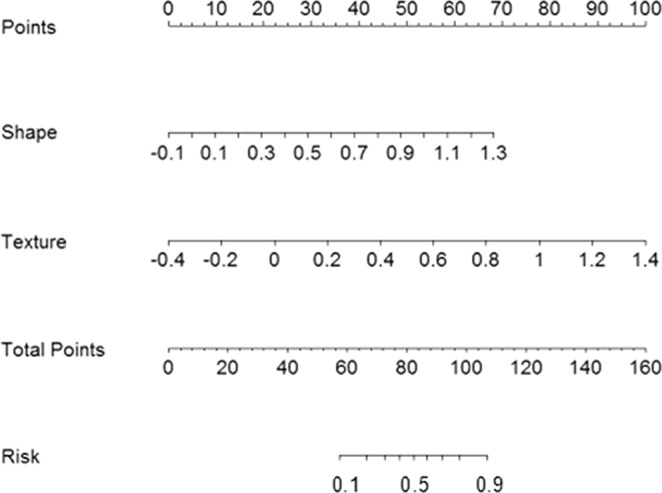
The nomogram for predicting the probability of axillary lymph node metastasis.

A nomogram for predicting ALNM was established in Fig. 6. The nomogram sets the scoring criteria according to the size of the regression coefficients of the morphological and texture features and gives each independent variable a score. For each patient, a total score can be calculated, and then the transfer function between the score and the probability of ALNM is used to calculate the probability of ALNM in each patient. The nomogram can better visualize the model intuitively.

## Discussion

Although breast cancer is the most common malignant disease among women, medical experts agree that the chances of survival are much higher with early detection^20^. With the continuous advancement of comprehensive treatment and breast cancer research, operation in breast cancer patients is gradually becoming minimally invasive. How to use the biological information of primary tumors to predict ALNM before surgery is a current topic at home and abroad^21^. Accurately predicting the status of ALNs is significant for axillary lymph node-negative breast cancer patients to avoid unnecessary ALND surgery and to reduce pain and costs.

In the study, we evaluated the diagnostic ability of traditional machine learning method combining with the radiomics features for preoperative prediction of ALNM. It is a quick and easily performed procedure, thereby saving time and cost for surgeons and pathologists. Studies have shown that, based on breast MRI, the SVM classifier has good discrimination ability for metastatic and nonmetastatic ALNs in breast cancer. Through the separate modeling and analysis of morphological features, texture features and each feature, the results showed that the combination of morphological features and texture features can obtain a better preoperative prediction model.

This study also has some limitations and inadequacies. First, the sample size of patients is small. Second, all subjects were patients from the First Hospital of China Medical University. The use of a multicenter dataset with different parameters may cause the model to behave differently. Large data sets from multiple centers should be studied to verify the robustness of our proposed radiomic model. The study of the prediction of ALNM based on breast MRI needs further study.

To select safe and effective treatment methods and reduce the occurrence of postoperative complications, it is very important to predict and diagnose ALN status. MRI can further evaluate the status of ALNM while detecting breast cancer, and it is an effective method to evaluate the status of ALN before surgery. It can provide valuable evidence for clinical operation and treatment and improve the diagnostic efficiency of doctors. In the future, more and better studies based on breast imaging to predict ALNM will provide a more reliable reference for clinicians and make greater contributions to the diagnosis, staging and treatment of breast cancer patients.

## Conclusion

This study provides a noninvasive and convenient method to facilitate the preoperative individualized prediction of ALNM in patients with breast cancer. Radiomic features of the primary tumor were extracted and analyzed using DCE-MRI with several machine learning methods. Looking forward to more and better researches based on breast imaging to predict ALNM, providing a more reliable reference for clinicians and making greater contributions to the diagnosis, staging and treatment of breast cancer patients.

## Supplementary information

Feature extraction

## Data Availability

The datasets generated during and/or analysed during the current study are available from the corresponding author on reasonable request.
