# Supplementary material for: Preoperative Prediction of Axillary Lymph Node Metastasis in Breast Cancer using Radiomics Features of DCE-MRI
Source: Sci Rep. 2019 Feb 19;9:2240. doi: 10.1038/s41598-019-38502-0 (PMC6381163; doi:10.1038/s41598-019-38502-0)
Supplement: Supplementary file 1 — Feature extraction [file 41598_2019_38502_MOESM1_ESM.pdf]

Preoperative Prediction of Axillary Lymph Node Metastasis in Breast Cancer using Radiomics Features of DCE-MRI

Xiaoyu Cui,[1]
Nian Wang,[1]
Yue Zhao,[1]
Shuo Chen,[1]
Songbai Li,[2]
Mingjie Xu,[1]
Ruimei Chai[*],[2]

[1]Northeastern University, Sino-Dutch Biomedical and Information Engineering School, Wisdom Street, Shenyang, China, 110819
[2]Radiology Department, The first hospital of China Medical University, 155# North Nanjing street, Heping district, Shenyang, China, 110001

Appendix 1

Feature extraction

In this study, a total of 58 candidate radiomics features were extracted. All feature extraction methods were implemented using MATLAB 2017a. The 58 features were divided into two types: morphological features, textural features.

Morphological features: In this group of features, we included 16 descriptors of the tumor region, including area, perimeter, roundness, centroid, the smallest rectangle containing the area of the mass, eccentricity of an ellipse with the same second-order moment as the mass area, diameter of a circle with the same area as the mass area, pixel ratio in both the lumps area and its smallest bounding rectangle, length of the long axis of the ellipse with the same second-order moment as the mass area (number of pixels), length of the short axis of the ellipse with the same second-order moment as the mass area (number of pixels), pixel ratio in the mass area and its smallest convex polygon, the angle of intersection between the x-axis and the long axis of the ellipse with the same standard second-order moment of the region, ratio of length to width, rectangles.

Textural features: Textural features are visual characteristics that reflect the homogeneity phenomenon of images and the arrangement of properties that change slowly or periodically on the body surface. It is represented by the grayscale distribution of the neighborhood of the pixel and its surrounding space. In the paper, textural features mainly included six types: the Gray-level co-occurrence matrix (GLCM), the Gray Level Run Length Matrix (GLRLM), Gray Level-Gradient Co-occurrence Matrix (GLGCM), Neighboring Gray-Level Dependence Matrix (NGLDM), grayscale histogram features, and Tamura features.

1) The GLCM is the matrix function that describes the distance and angle of each pixel. By calculating the correlation between two gray levels with certain

directions and distances, GLCM can reflect integrated information regarding the direction, interval, amplitude, and frequency of images. We extracted 8 radiomic features from the GLCM, mainly consisted of the mean and standard deviation of energy, entropy, contrast, and correlation. The features were as follows:

Energy: reflecting the uniformity of image gray distribution and texture thickness.

$$Asm = \sum_i \sum_j P(i,j)^2$$

Entropy: measuring the randomness of the image containing information and showing the complexity of the image. Entropy is greatest when all values in the co-occurrence matrix are equal or the pixel values exhibit maximum randomness.

$$Ent = -\sum_{i} \sum_{j} P(i，j) \log P(i,j)$$

Contrast: reflecting the clarity of the image and the depth of the texture. The texture groove is the deeper, the contrast is the greater and the visual effect the clearer; conversely, the contrast is small, the groove is shallow and the effect is blurred.

$$Con = \sum_i \sum_j (i-j)^2 P(i,j)$$

Correlation: also known as homogeneity, it measures the similarity of the gray level of an image in the row or column direction. Therefore, the value reflects the local gray correlation.

$$Corr = \frac{\sum_{i} \sum_{j} ((ij)P(i,j)) - u_x u_y}{\sigma_x \sigma_y}$$

2) The GLRLM is used to describe the distribution of pixel values. The GLRLM of an image reflects the comprehensive information of gray level about direction, adjacent interval and change range. The 5 GLRLM features mainly consisting:

Short run emphasis (SRE):

$$SRE = \frac{\sum_{i=1}^{N_g}\sum_{j=1}^{N_r}\left[\frac{\rho(i,j|\theta)}{j^2}\right]}{\sum_{i=1}^{N_g}\sum_{j=1}^{N_r}\rho(i,j|\theta)}$$

Long run emphasis (LRE):

$$LRE = \frac{\sum_{i=1}^{N_g}\sum_{j=1}^{N_r} j^2\rho(i,j|\theta)}{\sum_{i=1}^{N_g}\sum_{j=1}^{N_r}\rho(i,j|\theta)}$$

Gray level non-uniformity (GLN):

$$GLN = \frac{\sum_{i=1}^{N_g}\left[\sum_{j=1}^{N_r}\rho(i,j|\theta)\right]^2}{\sum_{i=1}^{N_g}\sum_{j=1}^{N_r}\rho(i,j|\theta)}$$

Run length non-uniformity (RLN)

$$RLN = \frac{\sum_{j=1}^{N_r}\left[\sum_{i=1}^{N_g}\rho(i,j|\theta)\right]^2}{\sum_{i=1}^{N_g}\sum_{j=1}^{N_r}\rho(i,j|\theta)}$$

Run percentage (RP):

$$RP = \sum_{i=1}^{N_g}\sum_{j=1}^{N_r}\frac{\rho(i,j|\theta)}{N_\rho}$$

$\rho(i,j|\theta)$ represents the gray run-length matrix; N_g represents the number of gray levels on an image; N_r represents the number of different runs on an image; N_p represents the number of pixels on an image.

3) The GLGCM adds gradient information into gray co-occurrence matrix, and integrates gray and gradient information of the image to get the better effect. The 15 GLGCM features mainly consisting: small gradient advantage, large gradient advantage, non-uniformity of gray distribution, non-uniformity of gradient distribution, energy, gray average, gradient average, the mean square deviation of gray, the mean square deviation of gradient, correlation, gray entropy, gradient entropy, mixed entropy, inertia, inverse moment.

4) The 5 NGLDM features mainly consisting: small number emphasis, large number emphasis, number non-uniformity, second moment, entropy.

5) The 6 grayscale histogram features mainly consisting:

Mean: a measure of the average brightness of texture.

$$\mathrm{m} = \sum_{i=0}^{L-1} \mathrm{z_i} P(z_i)$$

Variance: a measure of the average contrast of texture.

$$\sigma^2 = \sum_{i=0}^{L-1} (z_i - m)^2 P(z_i)$$

Third-order moment: a measure of the histogram skewness. For a symmetric histogram, this value is 0. If it is positive, the histogram is skewed to the right and if it is negative, the histogram is skewed to the left.

$$\mathrm{u_3} = \sum_{i=0}^{L-1} (z_i - m)^3 P(z_i)$$

Entropy: a measure of randomness. The greater the entropy, the greater the randomness and the greater the amount of information.

$$\mathrm{e} = -\sum_{i=0}^{L-1} P(z_i) \log_2 P(z_i)$$

Smoothness: a measure of the relative smoothness of texture brightness. For a region where the gray scale is uniform, the smoothness R is equal to 1, and for a region having a large difference in the value of the gray level, the R is equal to 0.

$$R = \frac{1}{(1+\sigma^2)}$$

Consistency: when all gray levels in the region are equal, the metric is the largest and starts to decrease from here.

$$U = \sum_{i=0}^{L-1} P^2(\mathrm{z_i})$$

L is the total number of gray levels, $\mathrm{z_i}$ is the first i gray level, $P(\mathrm{z_i})$ is the probability of the gray level $\mathrm{z_i}$ in the normalized histogram gray level

distribution, and $h(z_i)$ is the number of pixels whose gray level is z_i in the histogram.

6) Based on human psychology research on the visual perception of texture, Tamura et al. proposed the expression of texture features. The six components of the Tamura texture feature correspond to the six properties of the texture feature in terms of psychology, namely roughness, contrast, directionality, linelikeness, regularity, and roughness. In this study, roughness, contrast, and direction were used.

Appendix 2

The 38 features selected by LASSO include:

14 morphological features: perimeter, roundness, centroid, the smallest rectangle containing the area of the mass, eccentricity of an ellipse with the same second-order moment as the mass area, diameter of a circle with the same area as the mass area, pixel ratio in both the lumps area and its smallest bounding rectangle, length of the short axis of the ellipse with the same second-order moment as the mass area (number of pixels), pixel ratio in the mass area and its smallest convex polygon, the angle of intersection between the x-axis and the long axis of the ellipse with the same standard second-order moment of the region, ratio of length to width, rectangles.

1 NGLDM feature: second moment.

4 GLRLM features: short run emphasis, long run emphasis, run length non-uniformity, run percentage.

7 GLCM features: the mean and standard deviation of energy, entropy, contrast, and the standard of correlation.

10 GLGCM features: large gradient advantage, non-uniformity of gray distribution, energy, gradient average, the mean square deviation of gray, the mean square deviation of gradient, gradient entropy, mixed entropy, inertia, inverse moment.

1 Tamura feature: coarseness.

1 grayscale histogram feature: mean.
